# The role of immunohistochemistry in medullomyoblastoma – a case series highlighting divergent differentiation

**DOI:** 10.1186/1746-1596-3-18

**Published:** 2008-04-25

**Authors:** Man Updesh S Sachdeva, Mahesha Vankalakunti, Aruna Rangan, Bishan D Radotra, Rajesh Chhabra, Rakesh K Vasishta

**Affiliations:** 1 Department of Histopathology, Postgraduate Institute of Medical Education & Research, Chandigarh, India; 2 Department of Neurosurgery, Postgraduate Institute of Medical Education & Research, Chandigarh, India

## Abstract

**Aims:**

To analyse the histo-morphology of cases of medullomyoblastoma and identifying its divergent differentiation.

**Methods:**

A retrospective review of all cases reported as medulloblastoma between the period of Jan 2000 to Dec 2006 was carried out on Hematoxylin and eosin (H & E) stained slides. The cases were screened on light microscopy for primitive neuroectodermal component of a medulloblastoma accompanied by areas of "myoid" differentiation, identified on the basis of presence of strap cells (indicating a clear skeletal muscle differentiation) and/or large anaplastic cells with vescicular nuclei and moderate to abundant amount of eosinophilic cytoplasm. All these cases were subjected to a panel of immunohistochemical stains, including Desmin, GFAP, NFP, HMB45, SMA, S100, CK and EMA. Ultrastructral analysis was done on tissue obtained from paraffin blocks in 2 cases.

**Results:**

Male predominance (M:F = 5:1) was noted with an incidence of five percent of all cases of medulloblastoma (6 out of 120 cases) over a period of 6 years. Primitive neuroectodermal areas were accompanied with areas of "myoid" differentiation, 5 cases showing strap cells. Two cases with epithelial and cartilaginous differentiation were seen. Three cases showed focal melanocytic differentiation, identified only on HMB45 immunostaining. Four cases showed glial differentiation. Neuronal differentiation again was very focally seen in two cases, of which one was identified only by NFP immunostain. Seventh case is included in the study, however it is not considered to calculate incidence as it occurred beyond the period of 6 years of records search.

**Conclusion:**

Medullomyoblastoma is a rare childhood tumor of cerebellum. Majority of cases reveal divergent differentiation, which are identified with the help of panel of immunostains indicating multi-potential nature of primitive neuroectodermal cells.

## Introduction

Medullomyoblastoma (MMB) is a rare cerebellar embryonal neoplasm that occurs almost exclusively in children. It has a biphasic histo-morphology, containing myoblastic and primitive neuroectodermal components. MMB arises exclusively in the cerebellum. English literature provides some case reports of this rare tumor and an occasional series of small number of cases [1-11]. In the present series, we present histo-morphological features of 7 cases of medullomyoblastoma and try to find out any divergent differentiation by using a panel of immunostains.

## Materials and methods

A retrospective review of all cases reported as medulloblastoma over the period of 6 years in the department of Histopathology, Post graduate Institute of Medical Education and Research (PGIMER), Chandigarh, was carried out. Hematoxylin and eosin (H & E) stained slides as well as paraffin blocks of all these cases were available as archival material in the department. H & E slides were screened for cases showing primitive neuroectodermal component of a medulloblastoma accompanied by areas of "myoid" differentiation, identified on the basis of presence of strap cells (indicating a clear skeletal muscle differentiation) and/or large anaplastic cells with vescicular nuclei and moderate to abundant amount of eosinophilic cytoplasm. Morphological details on H & E stained slides of the selected cases were carefully noted, with a special mention to any other differentiations accompanying the two components of primitive neuroectodermal and rhabdomyoblastic cells. Sections from paraffin embedded tissues of all these cases were subjected to a panel of immunohistochemical stains, namely, Desmin, GFAP (Glial Fibrillary Acidic Protein), NFP (Neurofilament Protein), HMB45, SMA (Smooth Muscle Actin), S100, CK (Cytokeratin) and EMA (Epithelial Membrane Antigen). Ultrastructural analysis was done on tissue obtained from paraffin blocks in 2 cases.

## Results

A total of 120 cases of medulloblastoma were present in the retrospective search of files over the period of 6 years. A retrospective review of H & E stained slides showed 6 cases (5% of all medulloblastomas) which had morphological evidence of "myoid" differentiation accompanying the typical areas of primitive neuroectodermal cells. Three of these cases had been reported as medullomyoblastoma during routine reporting. The clinicopathological data of these cases have been summarized in Table 1. The case no. 7 that is included in the Table 1, was a previously diagnosed case of medullomyoblastoma, confirmed on ultrastructural analysis in the year 1993. However this case is beyond the period of 6 years of records search. This case was also subjected to same panel of immunhistochemical stains.

**Table 1:** Clinical data and histology of Medullomyoblastoma

**Case No.**	**Age**^#^**/Sex**	**Presentation**	**Duration of symptoms (days)**	**Imaging (CT/MRI)**	**Histopathological features**
1	28 y/M	Headache, vomiting, ataxic gait	14	Posterior fossa SOL	Predominant areas of PNE cells, nodules of cells showing smooth muscle differentiation, cartilaginous islands
2	6 mon/F	Vomiting, altered sensorium	15	Posterior fossa SOL, hydrocephalus	Predominant areas of PNE cells, RMB areas present, focal cartilaginous and epithelial differentiation
3	4 y/M	Recurrent headache, vomiting	20	Posterior fossa contrast enhancing SOL	Co-dominant RMB and PNE areas, many strap cells present
4	3 y/M	Headache, vomiting, altered sensorium	20	Posterior fossa SOL	Predominant areas of PNE cells, RMB areas present with strap cells
5	4 y/M	Persistant vomiting	60	Posterior fossa SOL, hydrocephalus	Predominant areas of PNE cells, RMB areas with strap cells present
6	8 y/M	Headache, vomiting, vertigo	60	Posterior fossa SOL	Predominant areas comprise of larger atypical cells with vesicular nuclei and moderate amount of cytoplasm, no typical strap cells present, rest of the areas show PNE cells
7	4 y/M	Vomiting, altered sensorium	30	Posterior fossa SOL	Predominant areas of PNE cells, RMB areas show strap cells

PNE – Primitive neuroectodermal; RMB – Rhabdomyoblastic; SOL – Space occupying lesion; Age^# ^y – years & mon-months

### Clinical characteristics

The patients ranged in age from 3 to 28 years (median age – 6 years). Six patients were male and one was female. The most common presentations were headache and vomiting, followed by altered sensorium, ataxia and vertigo (Table 1). In all patients, imaging revealed a posterior fossa space occupying lesion (SOL), involving the cerebellum.

### Pathology

At low power morphological features in all cases showed undifferentiated areas comprising of primitive neuroectodermal cells. These areas did not reveal definite rosette formation in any of the 7 cases. All the cases except Case 6 [Table 1] had interspersed hypocellular areas which on higher magnification showed features of rhabdomyoblastic differentiation. The cells with "myoid" differentiation had large eccentrically placed vescicular nuclei and moderate amount of eosinophilic cytoplasm. Strap cells, considered to be the best morphological indicator of skeletal muscle differentiation, were seen in all cases, except Case 1 and 6 [Table 1]. The areas with rhabdomyoblastic differentiation comprised of 20 to 50% of total tumor tissue.

**Figure 1:**
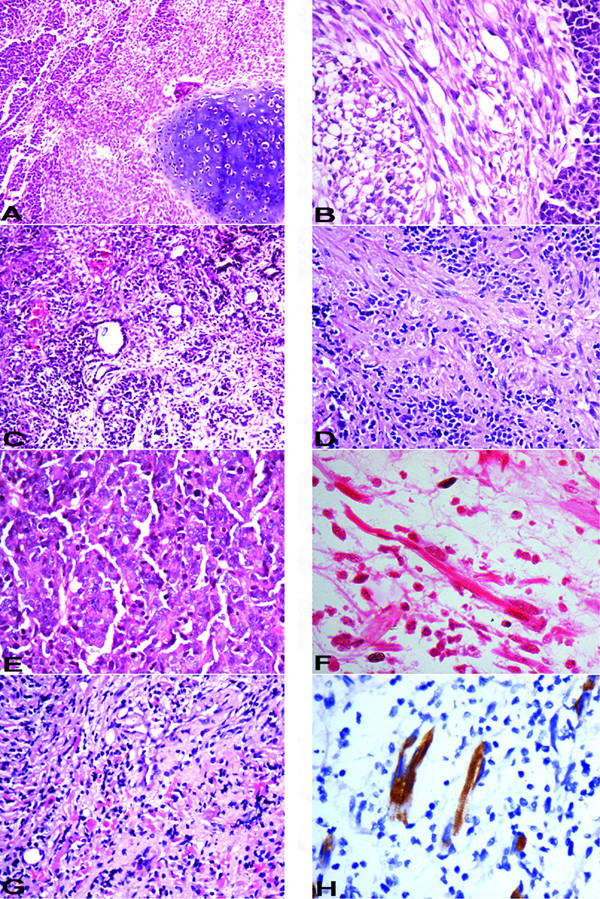
**A** – Areas of primitive neuroectodermal cells with islands of cartilage (10×, HE); **B** – Fascicular arrangement of spindle shaped cells with adjacent primitive neuroectodermal cells (20×, HE); **C** – Gland formation indicative of epithelial differentiation and focal myoid cells (10×, HE); **D** – Primitive neuroectodermal cells in fibrillary background with focal ganglionic differentiation (20×, HE); **E** – Larger "atypical" cells, with a vescicular nuclei, prominent nucleoli and moderate amount of cytoplasm (20×, HE); **F** – Strap cells (40×, HE); **G** – Admixture of primitive neuroectodermal cells and myoid differentiation of case 4 in Table 1 (10×, HE); **H** – Desmin immunostain highlighting striations in strap cells (40×, immunoperoxidase).

**Figure 2:**
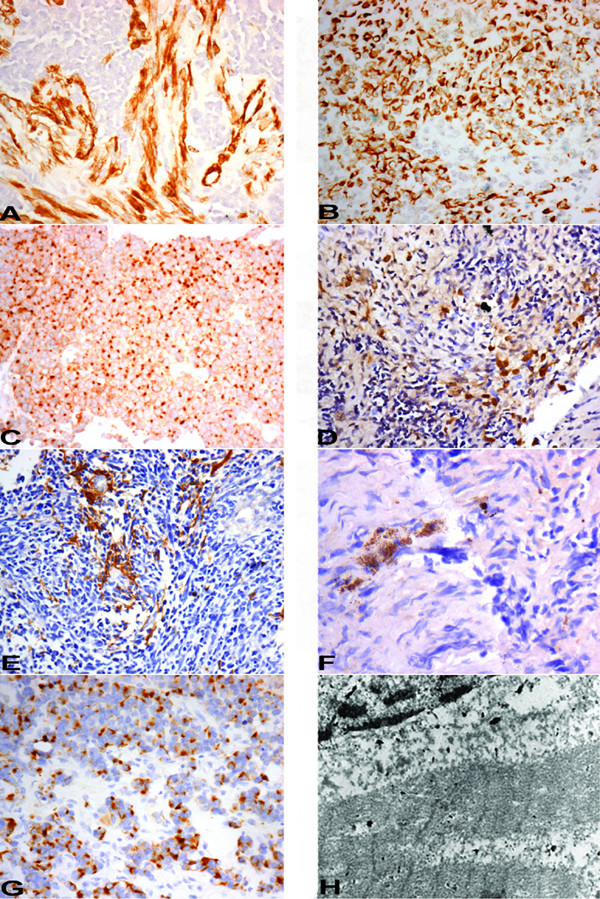
**A** – SMA immunostain expression in the spindle cells and vessel (internal control) along with absence of expression in primitive neuroectodermal cells (20×, immunoperoxidase); **B** – Cytokeratin immnostain positivity (20×, immunoperoxidase); **C** – EMA immunostain showing paranuclear dot positivity (20×, immunoperoxidase); **D** – HMB-45 immunostain showing focal cytoplasmic positivity (20×, immunoperoxidase); **E** – GFAP immunostain positivity in glial areas (20×, immunoperoxidase); **F** – NFP immunostain positivity in few cells (40×, immunoperoxidase); **G** – Desmin immunostain showing paranuclear dot positivity (20×, immunoperoxidase); **H** – Ultrastructure of strap cells showing prominent Z bands (10000×, Uranyl acetate with lead citrate).

Case 1 [Table 1] was an extremely rare case of medullomyoblastoma in adult patient, aged 28 years. On histopathological examination, the tumor predominantly had nodules of primitive neuroectodermal cells with areas of necrosis. Another prominent finding was presence of multiple islands of cartilage located in the centre of the primitive neuroectodermal nodules [Fig 1A]. Some of these nodules showed myoid differentiation comprising of large cells with vesicular nuclei and abundant pale cytoplasm. No striations however were noted in the cytoplasm of these cells. A fascicular pattern of arrangement was noted favouring leiomyomatous differentiation [Fig 1B]. Occasional focus however, did reveal cells with skeletal muscle differentiation. Desmin immunostain was positive in both of these foci. Smooth muscle actin [SMA] immunostain [Fig 2A], revealed a strong cytoplasmic positivity in the nodules confirming a smooth muscle differentiation of these cells. The chondrocytes showed strong nuclear positivity for S-100. Some of the undifferentiated cells surrounding cartilage also showed nuclear as well as cytoplasmic positivity with S-100. In addition focal positivity for HMB45, CK [Fig 2B], EMA, GFAP and NFP was also noted in small round cells. EMA positivity was in form of paranuclear dots [Fig 2C]. Overall, the tumor revealed muscle and cartilagenous differentiation identified on morphology. Immunostaining in addition revealed focal glial, neuronal, melanocytic and epithelial differentiation.

Case 2 [Table 1] predominantly had typical medulloblastic areas. Intervening areas of myoid differentiation were noted although typical strap cells were not seen. In addition there were 2 foci of cartilage formation, surrounded by the primitive neuroectodermal cells. An occasional focus in these cellular medulloblastic areas showed epithelial differentiation evidenced by the formation of gland like structures [Fig 1C]. Immunohistochemical staining revealed strong cytoplasmic positivity for desmin in the areas of rhabdomyoblastic differentiation. Focal collection of small round cells revealed a strong cytoplasmic granular positivity for HMB-45 indicating melanocytic differentiation [Fig 2D]. These cells were present in the loose stroma intervening typical medulloblastic areas, forming small clusters. The cells forming gland like structures revealed cytoplasmic positivity of cytokeratin. There were no areas of necrosis. Overall this tumor showed predominant areas of primitive neuroectodermal cells and rhabdomyoblastic areas accompanied with islands of cartilage; epithelial and melanocytic differentiation identified on immunohistochemistry.

Case 3 [Table 1] had predominant areas of rhabdomyoblastic differentiation comprising of many strap cells. Rest of the tumor had typical medulloblastic areas also accompanied by hypocellular fibrillary areas. Occasional neuronal differentiation was noted in the form of larger cell size, more abundant cytoplasm, vesicular nuclear chromatin [Fig 1D]. Desmin immunostain showed strong cytoplasmic positivity. The positivity was also seen in few undifferentiated cells adjacent to myoid areas. Coarse granular cytoplasmic positivity of HMB-45 was seen in few cells interspersed between nodules of primitive neuroectodermal cells. The hypocellular fibrillary areas were positive for GFAP [Fig 2E], nicely highlighting the cytoplasmic processes of the cells with glial differentiation. Small aggregates of cells in these fibrillary areas showed NFP positivity [Fig 2F] indicating neuronal differentiation.

Case 6 [Table 1] was diagnosed as medulloblastoma and did not have any areas typical of rhabdomyoblastic differentiation; however, there were many larger "atypical" cells, with a vescicular nuclei, prominent nucleoli and moderate amount of cytoplasm, giving an epithelioid appearance [Fig 1E]. On immunohistochemistry, most of these atypical larger cells showed paranuclear dot positivity for desmin [Fig 2G]. A few cells also showed GFAP positivity.

Case 7 [Table 1] was diagnosed as medullomyoblastoma, and confirmed by electron microscopy. The strap cells in the areas of myoid differentiation, showed presence of striations in some of these cells [Fig 1F]. Immunostains performed revealed strong desmin positivity in strap cells and in few cells it nicely highlighted striations in the cytoplasm [Fig 1H].

Case 1 and 3 [Table 1] were subjected to ultrastructural analysis. The tissue was obtained from paraffin embedded blocks. Both the cases showed presence of alternating thin [actin] and thick [myosin] filaments in a parallel arrangement with distinct Z-banding in several places [Fig 2H].

All these cases had a varied percentage of differentiating cell components formed by the primitive neuroectodermal cells. These were accompanied with morphologically identifiable areas of "myoid" differentiation, 5 cases showing strap cells, a hallmark of rhabdomyoblastic differentiation. Two cases with epithelial and cartilaginous differentiation were seen. Three cases, in addition, showed focal melanocytic differentiation, identified only on HMB45 immunostaining. Glial differentiation was more easily picked up on H & E stained slides, in 4 out of 7 cases, by the presence of loose fibrillary areas, containing larger, differentiated cells. Neuronal differentiation again was very focally seen in 2 cases, identified only on NFP immunostaining. Two cases revealed only myoid differentiation accompanying typical medulloblastoma.

## Discussion

Medullomyoblastoma was described first by Marinesco and Goldstein in 1933 [1]. Since then there has been only few case reports of this rare embryonal tumor [2-9], and an occasional case series [10,11]. We present series of 7 cases, 6 of which have been seen over a period of 6 years, amounting to 5% of all medulloblastomas reported during this period. The tumor was seen in early childhood, except for one adult male patient, aged 28 years. There are only 3 case reports of medullomyoblastoma in adults [11-13]. This tumor showed smooth muscle, skeletal muscle and cartilaginous differentiation accompanied with focal melanocytic, glial, neuronal and epithelial differentiations. There was a clear morphological evidence to suggest that the primitive neuroectodermal cells are actually differentiating into muscle and cartilaginous elements. The other differentiations were focal and not well appreciated on routine H & E stained slides. These were identified only with the help of immunostains. This case also strongly supports the idea of the multipotential nature of the primitive neuroectodermal cells. There are some case reports of differentiations in a medulloblastoma other than muscle and these include melanocytic, cartilaginous and epithelial differentiations [6,14,15]. In addition, melanocytic rhabdomyomedulloblastomas have also been rarely reported [16]. In the present series a panel of immunostains has established the presence of varied differentiations which accompany the myoid differentiation in medullomyoblastoma. There were 2 cases [Case 1 and Case 4, Table 1] which had accompanying cartilaginous islands. Both of these cases showed focal epithelial and melanocytic differentiations. The pattern of desmin immnostaining has also been of interest, as in most of the cases it showed diffuse cytoplasmic positivity, except in Case 6 [Table 2], where it was seen as a paranuclear dot and in Case 7 [Table 2], the immunostain highlighted cytoplasmic striations in the strap cells.

**Table 2:** Immunohistochemistry profile of Medullomyoblastoma

	**Immunohistochemistry**							
	
**Case No.**	**Desmin**	**HMB-45**	**GFAP**	**NFP**	**SMA**	**S100**	**CK**	**EMA**
1	+	+	+	+	+	+	+	+
2	+	+	-	-	-	+	+	+
3	+	+	+	+	-	-	-	-
4	+	-	-	-	-	-	-	-
5	+	-	-	-	-	-	-	-
6	+	-	+	-	-	-	-	-
7	+	-	+	-	-	-	-	-

A differential diagnosis in such cases of posterior fossa tumor is an atypical teratoid/rhabdoid tumor [AT/RT]. However, this is mostly seen in early infancy and preferentially located in cerebellar hemispheres, rather than arising from vermis. Histologically, the tumor cells are polygonal with abundant eosinophilic cytoplasm and eccentric nuclei and on immunostaining, these cells are variably positive for desmin, vimentin, GFAP, CK, EMA, SMA and synaptophysin [17].

Medullomyoblastomas, have been treated by a combination of surgery and/or chemotherapy and/or radiotherapy [10]. Most of the cases reported in the literature had a poor outcome. Three out of 6 cases, reported by Helton et al, died of the disease within 2 years [10]. One patient, however, remained free of disease for above 15 years, following treatment of a recurrence. Another case report of medullomyoblastoma in the literature has mentioned a long survival for over 11 years without recurrence [18]. All patients in our series underwent partial or total tumor resection with radiotherapy and/or chemotherapy. Most of the patients were lost to follow up at varied time intervals and hence the data on follow up remains unsatisfactory.

Present series of medullomyoblastoma identifies it to be a tumor of early childhood, with only one exceptional case presenting in adulthood. There is also a clear male predominance [M:F = 6:1]. Five percent of all cases of medulloblastoma [6/120], over a period of 6 years, showed myoid differentiation, the incidence being higher than in the series by Helton et al, which reported it to be about 3% of all patients of medulloblastoma [10]. The cases in our series showed a variety of differentiations; including cartilaginous, epithelial, glial, neuronal, melanocytic differentiations, most identified only on immunohistochemistry. Hence, this study also highlights the multipotential nature of primitive neuroectodermal cells of medulloblastoma and utility of these immunostains in highlighting the divergent differentiation.

## Competing interests

The authors declare that they have no competing interests.

## Authors' contributions

MSS and MV contributed to study design, collecting data, analysis and writing the manuscript. AR contributed to the collecting data. BDR, RC and RKV contributed in interpretation, and in deciding to submit the manuscript for publication.
